# Chromosomal inversion polymorphisms shape human brain morphology

**DOI:** 10.1016/j.celrep.2023.112896

**Published:** 2023-07-27

**Authors:** Hao Wang, Carolina Makowski, Yanxiao Zhang, Anna Qi, Tobias Kaufmann, Olav B. Smeland, Mark Fiecas, Jian Yang, Peter M. Visscher, Chi-Hua Chen

**Affiliations:** 1 Center for Multimodal Imaging and Genetics, University of California San Diego, La Jolla, CA 92093, USA; 2 Ludwig Institute for Cancer Research, La Jolla, CA 92093, USA; 3 School of Life Sciences, Westlake University, Hangzhou, Zhejiang 310024, China; 4 Westlake Laboratory of Life Sciences and Biomedicine, Hangzhou, Zhejiang 310024, China; 5 Department of Psychiatry and Psychotherapy, Tubingen Center for Mental Health, University of Tübingen, 72076 Tübingen, Germany; 6 Norwegian Centre for Mental Disorders Research, Oslo University Hospital and University of Oslo, 0450 Oslo, Norway; 7 Division of Biostatistics, University of Minnesota School of Public Health, Minneapolis, MN 55455, USA; 8 Institute for Molecular Bioscience, The University of Queensland, Brisbane, QLD 4072, Australia; 9 Lead contact

## Abstract

The impact of chromosomal inversions on human brain morphology remains underexplored. We studied 35 common inversions classified from genotypes of 33,018 adults with European ancestry. The inversions at 2p22.3, 16p11.2, and 17q21.31 reach genome-wide significance, followed by 8p23.1 and 6p21.33, in their association with cortical and subcortical morphology. The 17q21.31, 8p23.1, and 16p11.2 regions comprise the *LRRC37, OR7E,* and *NPIP* duplicated gene families. We find the 17q21.31 *MAPT* inversion region, known for harboring neurological risk, to be the most salient locus among common variants for shaping and patterning the cortex. Overall, we observe the inverted orientations decreasing brain size, with the exception that the 2p22.3 inversion is associated with increased subcortical volume and the 8p23.1 inversion is associated with increased motor cortex. These significant inversions are in the genomic hotspots of neuropsychiatric loci. Our findings are generalizable to 3,472 children and demonstrate inversions as essential genetic variation to understand human brain phenotypes.

## In brief

Wang et al. demonstrate that inversion polymorphisms are located within the genomic hotspots of neuropsychiatric loci and are linked to brain morphology. This study emphasizes that inversion polymorphisms, along with other genetic variants, play a significant role as contributors to the phenotypic diversity observed in brain development and disorders.

## Graphical Abstract

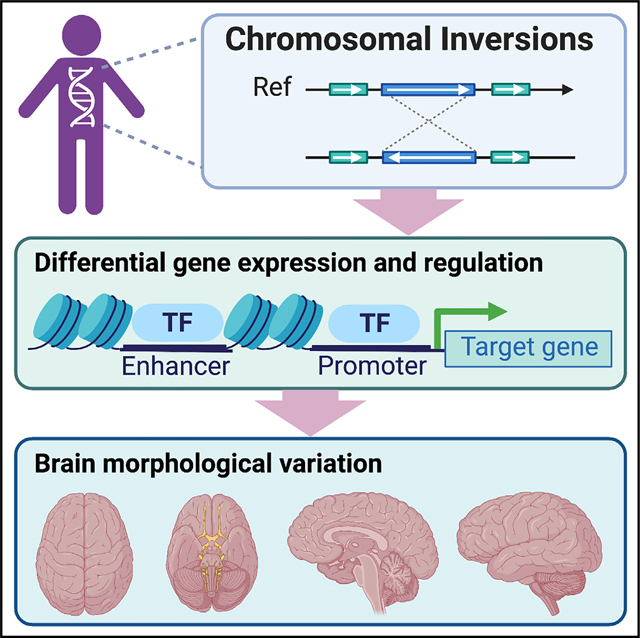

## INTRODUCTION

Polymorphic chromosomal inversions are common structural variations in which a chromosomal segment is in the reversed orientation.^1,2^ Recent work identified 729 inversions in humans and an average of 117–156 inversions per human genome.^3–5^ The inversions included in this study span from less than 1 Kbps to 4 Mbps, and their reported frequencies may range from about 6% to over 70% in various populations.^6^ Significant geographic- and ethnicity-related variation in frequencies has been observed in several inversion regions, such as the 17q21.31 inversion (17q21.31-inv), which is most common in the European population (~20%), particularly in the Mediterranean region (~30%), but is nearly absent in South and East Asia.^7^ Nearly 60 inversions intersect with regions of microdeletion and microduplication syndromes.^4,5^ These findings suggest that inversions constitute a substantial source of genetic variation for diseases and normal phenotypes. A small fraction of these inversions can be robustly characterized using genotype-array data through their impact on linkage disequilibrium (LD) patterns, leaving a detectable signature by principal-component analysis in inversion-calling algorithms and allowing for testing associations between inversions and phenotypes.^8,9^ In this study, we use the term “inversion” to refer to an inversion region, an inverted variant/allele (haploid), or an inverted genotype (diploid).

Large inversions are often flanked by segmental duplications (SDs) (repeats with ≥90% sequence identity) or mobile elements (e.g., *LINE1* or *Alu*) at inversion breakpoints.^3,10,11^ The hominid lineage has experienced a burst of SDs, which constitute ~7% of the human genome. SDs contribute disproportionately to genetic variation within and between ape species and play an important role on human-specific diseases and traits.^12,13^ Human SDs are gene-rich repeats, and gene duplication is recognized as a source of evolutionary innovation.^14^ The *LRRC37, NPIP* (nuclear pore interacting protein), and *OR7E* gene families located at 17q21.31-inv, 16p11.2-inv, and 8p23.1-inv, respectively, are among the 14 core duplicons previously identified to have expanded in the hominid lineage.^15,16^ There is some evidence to suggest that these gene families are associated with increased brain size across primates,^17^ although further replication is needed. Inversions suppress recombination leading to extended LD blocks and segregation of haplotype families, which provides a unique lens into the genomic impact of these complex structural variation regions on human phenotypic variation.

Although human and great ape species have ~98% sequence similarity, inversions are the most common chromosomal rearrangements that differentiate the genomes of the two species.^18^ In addition to differences between species, inversions are also important for phenotypic variations in humans, and their impact on brain-related phenotypes remains underexplored.^9,19^ Previous evidence showed an association between the 8p23.1-inv and neuroticism.^9,20^ Without using inversion calling, genetic variants at 17q21-inv were uncovered by genome-wide association studies (GWASs) of cortical and subcortical structures^21,22^ or by haplotype analysis of the *MAPT/tau* gene at 17q21.31 linked to risk of various neurodegenerative disorders.^23–27^

Compared to pathogenic copy-number variants (CNVs), common inversion polymorphisms are typically a balanced rearrangement, rather than a loss, of genomic materials and are more prevalent in the population. This facilitates the investigation of inversions within population-based databases, such as the UK Biobank (UKB) and Adolescent Brain Cognitive Development (ABCD) study. We previously observed genetic variants at the 8p23.1-inv and 17q21.31-inv associated with cortical morphology.^28^ Here, we present a comprehensive association analysis on 17 common inversions implemented in an inversion-calling method, *scoreInvHap*,^6^ to study their links to morphometrics derived from neuroimaging. Furthermore, the new high-throughput genotyping method characterizes ~45 common inversions, some of which can be tagged by single-nucleotide polymorphisms (SNPs).^19^ We utilize these tag SNPs to expand the list of the inversions in this study. For significant associations, we investigated expression patterns of genes at the inversion regions across developmental time points from the BrainSpan Atlas.^29^ We further focus on the most significant association, namely 17q21.31-inv, to uncover its genetic impact to study how the 17q21.31-inv could lead to alterations of gene expression, methylation, and isoform splicing.^30,31^

## RESULTS

### SNPs at 17q21.31-inv play a key role in cortical morphology

**Figure 1.**
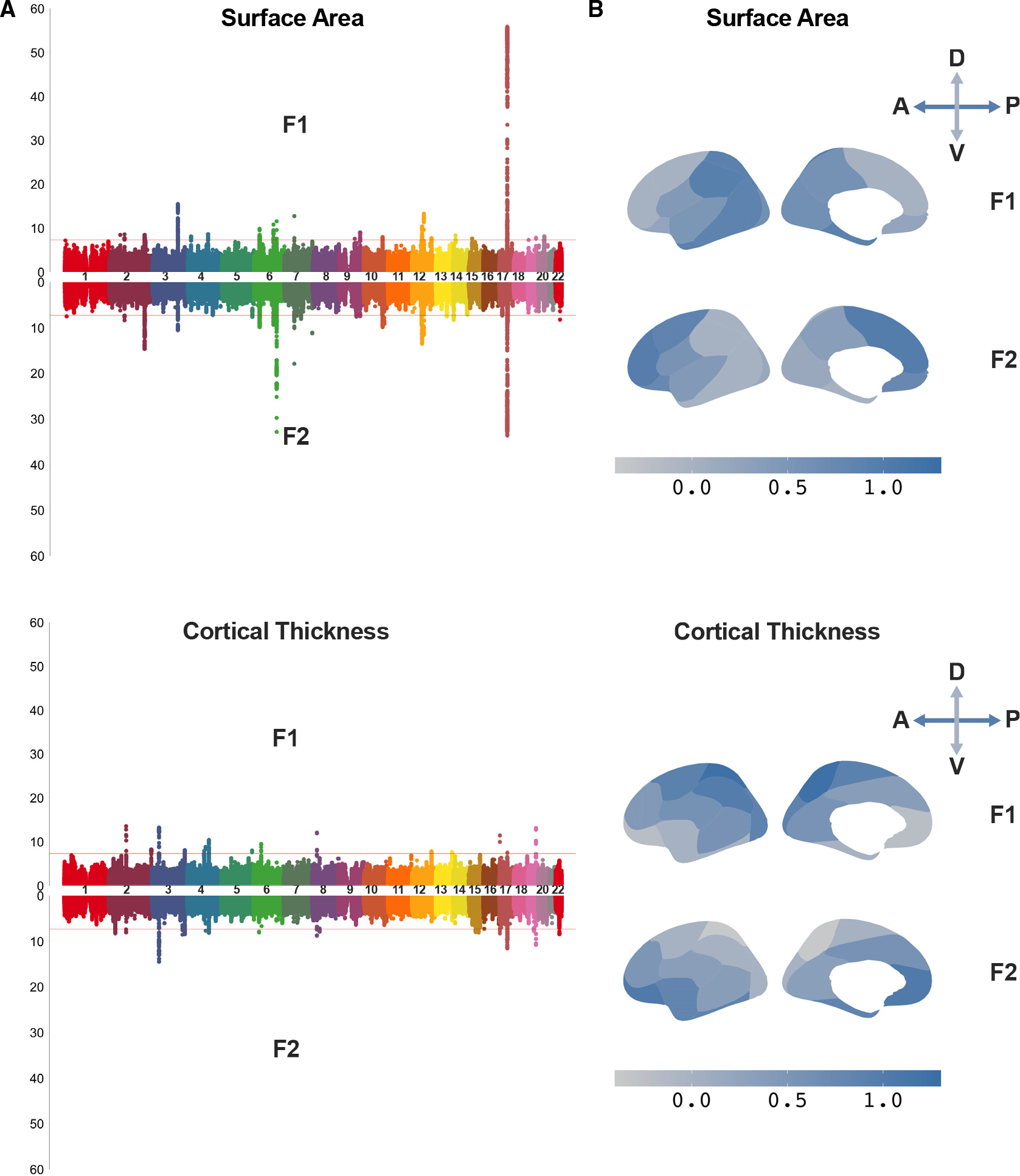
Genetic latent factors underlying cortical morphology point to inversion polymorphisms (A) Miami plots of SNP association with each salient latent factor of the two-factor models estimated by genomic SEM for area and thickness separately. Genetic latent factors (F1 and F2) represent major axes of genetic variation underlying all cortical regions. The strongest association signals between the latent factors and the genetic variants reside in the 17q21.31 inversion region for area (p < 10^−56^), whereas there are more widespread effects across the genome such as from chromosomes 3 and 17 for thickness. Genome-wide significance line represents p = 5 × 10^−8^. (B) Brain maps show standardized effects of each latent factor on each brain region, adapted from Makowski et al.^28^ The two latent factors recapitulated the anterior-posterior (A-P) and dorsal-ventral (D-V) gradations of cortical patterning for area and thickness, respectively.

In our previous work, we identified the salient latent factors underlying 24 regions of cortical surface area and thickness parcellated according to our genetically informed atlas (Table S1).^32,33^ This was conducted with a two-factor genomic structural equation model (SEM), as informed by the highest cumulative variance explained and model fit indices (see genomic structural equation modeling in STAR Methods), using autosomal variants of 34,720 adults from the UKB cohort of European ancestry.^28^ These genetic latent factors represented pleiotropic effects on morphometric variations across the cortex and pointed to 17q21.31-inv as a key contributor to individual size variations on cortical morphology. We showed a convergence of common SNPs and inversions where 1,627 SNPs mapped to 17q21.31-inv were found to be significantly associated with the above-mentioned latent factors (Figure 1). These findings prompted us to estimate the effect sizes of multiple inversions to elucidate their influence on the human brain.

### Inversion calling yields expected allele frequencies

To investigate the impact of inversion polymorphisms on cortical morphology, we included the variants of both autosomes and the X chromosome and performed inversion calling in our sample of 34,720 adults (Table S2). Out of the 21 inversions that can be classified by *scoreInvHap*, we excluded three that were not yet experimentally validated according to the Human Polymorphic Inversion database^34^ or the Human Genome Structural Variation Consortium (HGSVC) (Table S3)^4^ and one with low result quality (only 73.7% passed the two quality control [QC] steps) (Table S3; see STAR Methods). After removing 859 related subjects (genetic relatedness > 0.1), 33,861 participants were included in a multiple linear regression analysis (Table S3). The *scoreInvHap* method identifies individuals as having one of three different haplotype genotypes based on inverted (I) or non-I (NI) state, I/I, N/I, and N/N, and coded as 2, 1, and 0 by the dose of the I allele. All of our inversion genotypes showed good correspondence to the expected allele frequencies in the reference genome (Table S3). To further test the accuracy of our inversion calling, we classified the 17q21.31-inv status of 17 available HGSVC subjects by *scoreInvHap* and found that they were highly consistent with those reported by the HGSVC based on a multiplatform sequencing approach (Table S3). We also included 26 additional inversions with their genotypes called based on their reported good tag (LD r^2^ > 0.9) SNPs in the European population.^19^ Eight inversions (HsInv0004 [1q31.3], HsInv0040 [2q22.1], HsInv0045 [21q21.3], HsInv0058 [6p21.33], HsInv1053 [7q11.22], HsInv 0092 [6q23.1], HsInv0501 [8p23.1], and HsInv0573 [17q21.31]) were also included in *scoreInvHap*, and the results from *scoreInvHap* and based on tag SNPs were highly consistent with accuracies of 86.69%–99.98% (Table S3).

### Conspicuous effect of the 17q21.31-inv on global brain morphometry

**Figure 2.**
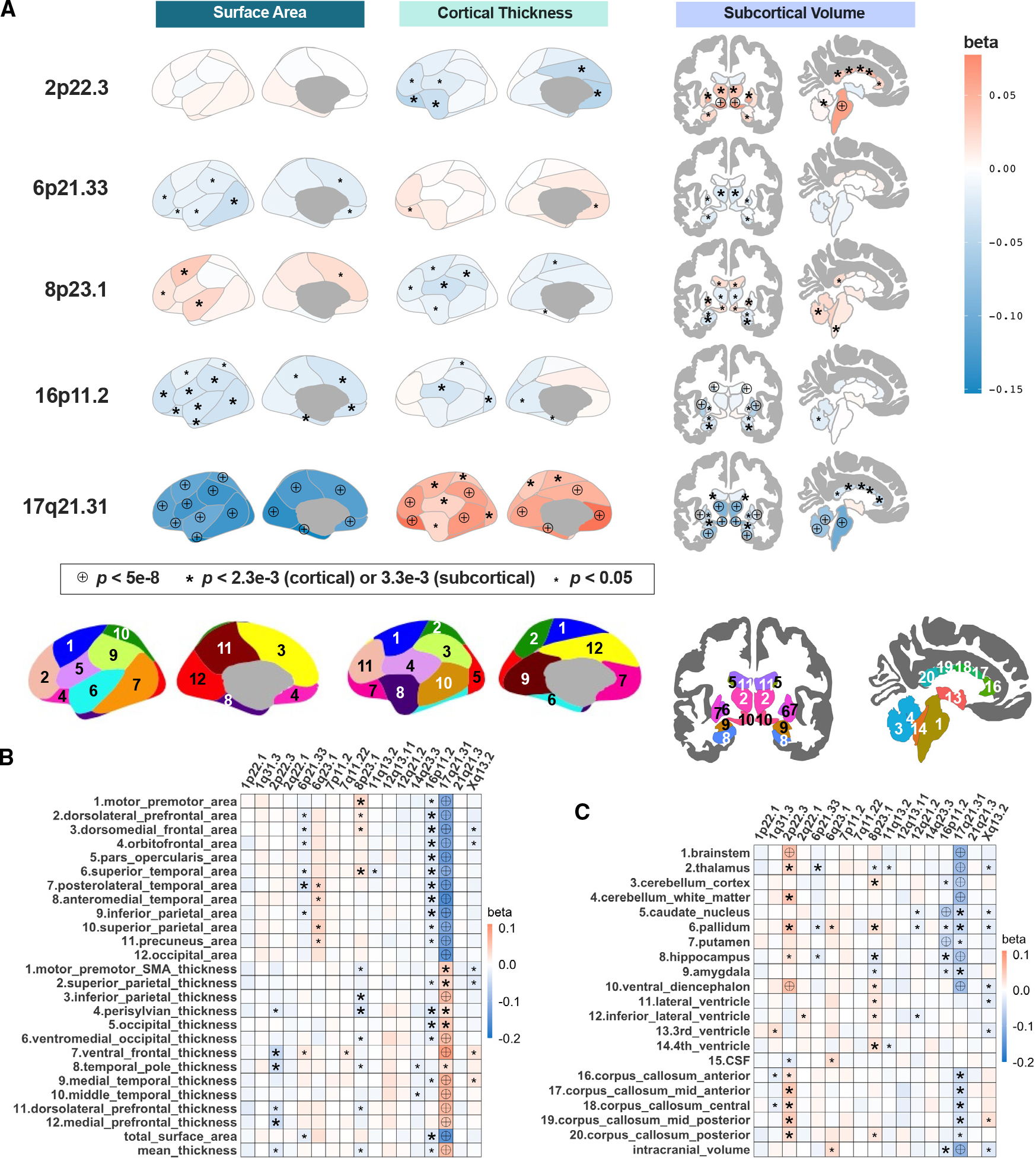
Genetic effects of inversions on cortical and subcortical structures (A) Brain maps highlighting the spatial patterns of genetic associations with morphometric measures for five significant inversions. The bottom row depicts the brain atlases. Numbering of regions follows labels shown on the heatmaps in the bottom panels. (B–C) Heatmaps of associations colored according to the beta coefficients of the regression models for all comparisons (left: cortical; right: subcortical structures). Labels denote nominal (small asterisks), Bonferroni-corrected (big asterisks), and genome-wide significance (encircled crosses). See Figures 1 and 2 and Table S4 for inversion association results with regional brain morphology when global brain size is taken into account.

The strongest associations emerged with the 17q21.31-inv with decreased total surface area and modestly increased cortical thickness (p < 2.3 × 10^−3^, lowest p = 6.3 × 10^−65^, where p < 0.05/*t*_e_, with *t*_e_ = 22 being the effective number of independent phenotypes^35,36^). The effects of the 17q21.31-inv are relatively large compared to GWAS signals of common polygenic phenotypes (~0.01% variance explained),^37^ where we found the largest association between this inversion and a decreasing anteromedial temporal area (0.86% variance explained). Furthermore, the 2p22.3-inv, 8p23.1-inv, and 16p11.2-inv were related to multiple cortical phenotypes, among which the 16p11.2-inv was also related to reduced total surface area (p = 2.1 × 10^−4^; Figure 2; Table S4).

We further studied 20 subcortical and intracranial volumes based on the *aseg* atlas in FreeSurfer (Table S1).^38^ Again, we observed strong associations between the 17q21.31-inv and reduced intracranial volume (p = 3.1 × 10^−26^, *t*_e_ = 15; Figure 2; Table S4). The same 2p22.3-inv, 6p21.33-inv, 8p23.1-inv, and 16p11.2-inv were linked to one or more subcortical volumes (lowest p = 2.2 × 10^−8^, 2.9 × 10^−3^, 4.2 × 10^−4^, and 3.5 × 10^−15^, respectively). The effects of 16p11.2-inv on the decreases in caudate and putamen volume explained ~0.1%–0.2% of variance.

To assess how well our results generalize to an independent sample, we performed an additional analysis to generalize the above inversion association analysis to a relatively small cohort of 3,472 children from the ABCD study. We observed significant consistency between the results from the UKB discovery and ABCD generalization cohorts, with inversion effect correlation estimates of 0.82 (p = 1.4 × 10^−11^) for cortical area phenotypes and 0.83 (p < 2.2 × 10^−16^) for subcortical phenotypes, accounting for errors in estimated inversion effects, as well as sign concordance rates of 0.88 (p = 2.5 × 10^−7^) for cortical area phenotypes and 0.79 (p = 1.1 × 10^−6^) for subcortical phenotypes. In contrast, we found no significant beta correlations between UKB and ABCD for the cortical thickness phenotypes. Interestingly, while the 17q21.31-inv was associated with increased thickness in UKB, this association was absent in ABCD (Table S4; Figures S2 and S3). The 17q21.31-inv has been previously identified as a protective variant for neurodegenerative diseases, and it is possible that carriers of this inversion experience less thickness decline over their lifespan. However, the effects of inversions on thickness are not as strong as those on surface area, which may partially contribute to the observed lack of correlation. More in-depth studies are needed to understand how this genetic variant influences cortical thickness in different age groups.

### Autosomal and X chromosomal inversions associated with regional brain morphology

**Figure 3.**
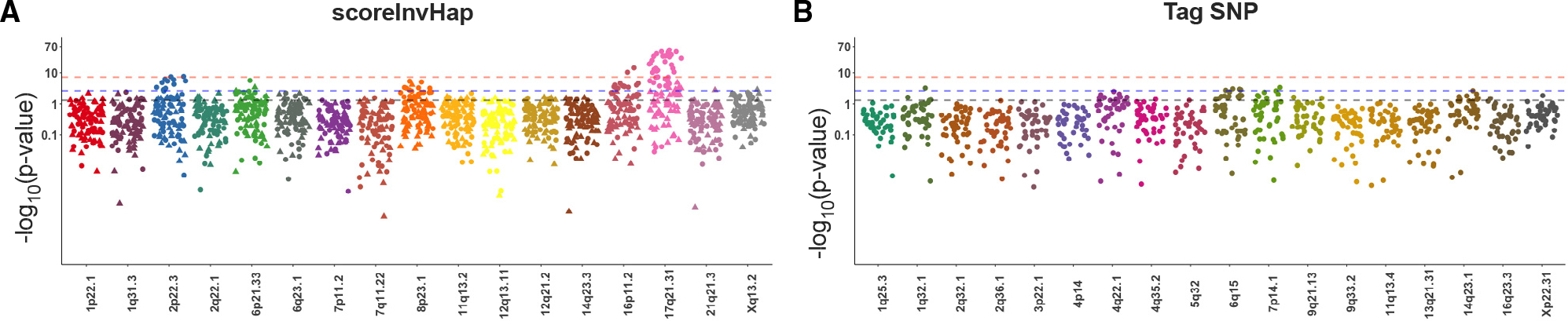
Manhattan plots visualize the association results for each individual inversion-morphology pair using both *scoreInvHap* and tag SNP approaches The dotted lines indicate the significant levels, ranging from genome-wide (p < 5 × 10^−8^), Bonferroni-corrected (p < 2.3 × 10^−3^), to nominal significance (p < 0.05). For the inversions identified through *scoreInvHap* (A), several significant inversion-morphology pairs exceeded the Bonferroni-corrected significance level. These significant pairs also showed good generalizability between the UKB and ABCD cohorts, as indicated by the symbols used (circles for UKB and triangles for ABCD). Specifically, three inversions (2p22.3, 16p11.2, and 17q21.31) reached genome-wide significance in the UKB cohort. Two additional inversions (6p21.33 and 8p23.1) reached nearly genome-wide significance. See Figure S3 and Table S4 for results after adjusting for global measures.

To further assess the impacts of inversions on regional measures, we repeated the above association analyses after adjusting for global measures by preresidualization (e.g., total surface area or mean cortical thickness). This analysis aimed to demonstrate the contributions of inversions to regional variations that have disproportionately larger effects relative to the entire brain. Thus, a positive association with frontal surface area would represent a larger increase relative to global expansion, as was the case for 8p23.1-inv (lowest p = 1.1 × 10^−7^), or a smaller decrease relative to global reduction, as was the case for 17q21.31-inv (lowest p = 4.4 × 10^−11^). Overall, significant associations of 2p22.3-inv, 6p21.33-inv, 8p23.1-inv, 11q13.2-inv, 16p11.2-inv, 17q21.31-inv, and Xq13.2-inv were demonstrated with cortical regions (*t*_e_ = 22; Figures 3, S1, and S3; Table S4), and 2q22.3-inv, 8p23.1-inv, 17q21.31-inv, and 16p11.2-inv were linked to subcortical structures (*t*_e_ = 15; Figures 3, S1, and S3; Table S4).

### Distinct spatial gradients of inversion-morphology association patterns across the cortex

We observed distinct spatial distribution patterns of significant inversion-morphology associations across the cortex, with their effect sizes following an anterior-posterior (A-P), ventral-dorsal, or medial-lateral gradient. For example, the 17q21.31-inv was associated with graded reductions in surface area along the A-P axis, with the greatest reduction in occipital and anteromedial temporal surface areas, accompanied by modest graded increases in thickness, with the largest increase in the orbitofrontal cortex (Figure 2). The Xq13.2-inv was related to increased rostral but decreased dorsal cortical thickness after adjusting for global measures (Figure S1).

### Convergent effects of the inversions and SNPs at inversion regions

We performed conditional analysis using GCTA-COJO to evaluate the significant inversion regions and found the association signals of the inversions and the SNPs within these genomic regions to be highly overlapping. These significant inversions were well tagged by SNPs. One extreme case is 17q21.31-inv, where at least ~100 SNPs are found to be in perfect LD with the inversion and any of them can tag the inversion as indicated previously.^7,39^ This is in line with our previous GWAS on cortical morphology in which we identified significant SNPs that mapped to genes located within the 17q21.31-inv, 8p23.1-inv, and 16p11.2-inv regions.^28^ Thus, although inversions do not add more variance explained than those already discovered in GWASs, identifying inversion associations could aim at understanding the underlying genetic mechanisms of these GWAS signals and at designing more effective follow-up experiments given that inversions alter genomic structure and could disrupt regulatory architecture more so than individual SNPs.^31,40^

### Developmental changes in the expression of inversion-related genes

**Figure 4.**
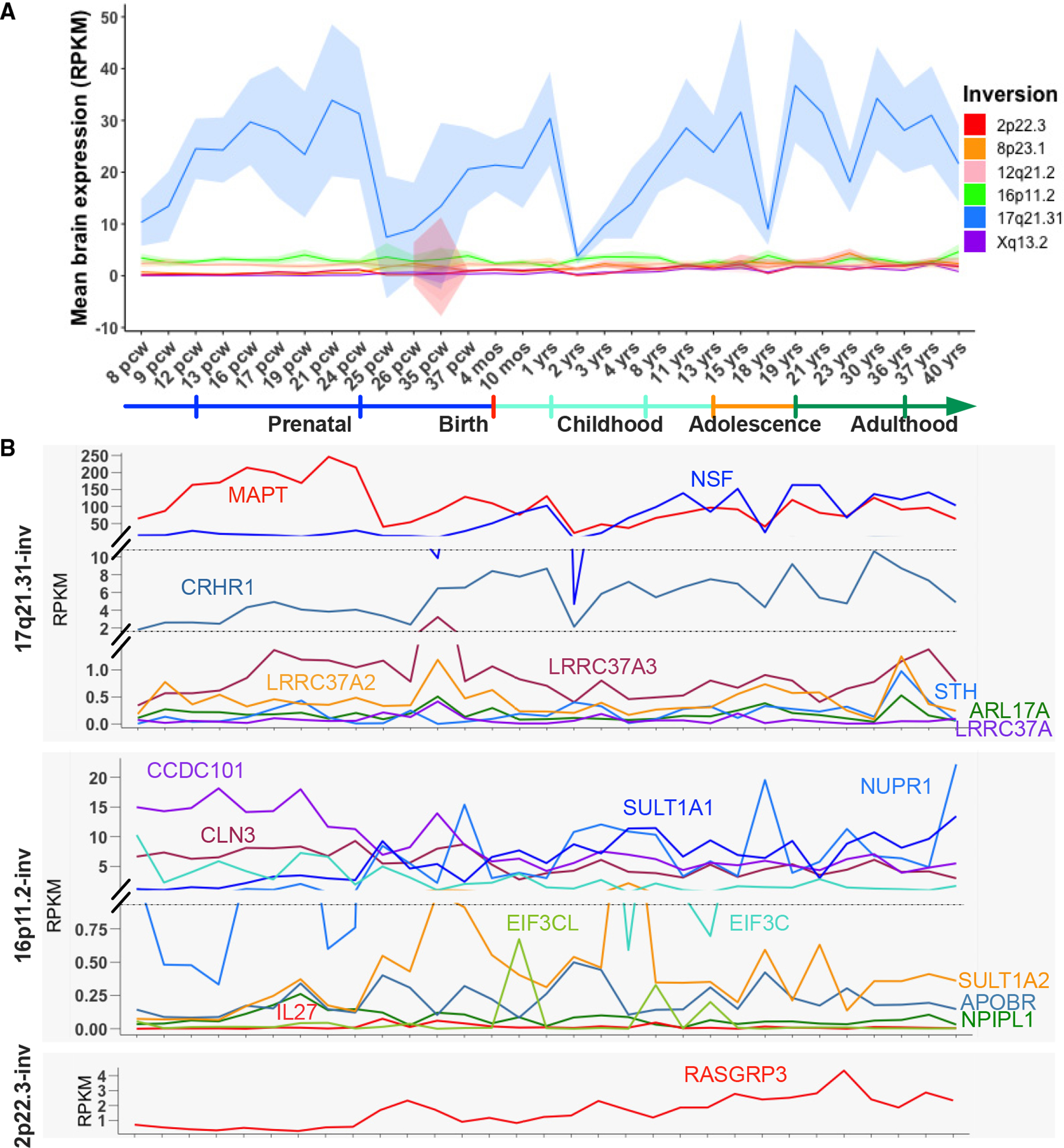
Expression profiles of genes located within the inversion regions across developmental time points (A) Mean expression levels of genes located within the inversion regions are plotted across time for the inversions that showed significant association with brain morphology. Shaded parts represent 95% confidence intervals. (B) The line graphs show the expression of individual genes within the 2p22.3 inversion (2p22.3-inv), 16p11.2-inv, and 17q21.31-inv, the three inversions that were identified to be associated with brain morphology with genome-wide significance (p < 5 × 10^−8^). Because these genes are expressed at very different ranges, we insert breaks on the y axis to give the full range of expression levels of all the genes. Each line shows a different gene, and each point shows a different developmental time point. Heatmaps for the 8p23.1-inv, 12q21.2-inv, and Xq13.2-inv are provided in Figure S4. We did not plot the remaining inversions from *scoreInvHap* since they do not overlap with any genes. Only genes present in the BrainSpan Atlas are displayed. pcw, post-conception week(s); yo, year(s) old.

Next, we explored the importance of genes within inversions on brain structure through their expression patterns from prenatal life to adulthood (Figures 4 and S4). We obtained gene expression data from the BrainSpan Developmental Transcriptome in the form of reads per kilobase million (RPKM) values.^41^ Notably, we observed substantially high mean expression levels for genes located in the 17q21.31-inv region (Figure 4A). Upon further analysis, we found that *MAPT* and *NSF* were primary drivers of this high expression (Figure 4B). These genes are known to play a critical role in maintaining normal synaptic function and have been linked to neurodegenerative disorders, autism, and anxiety.^42–44^ Specifically, *MAPT* was highly expressed at the mid-late prenatal stage, which corresponds to the phases of neurogenesis, neuronal migration, and early synapto- and gliogenesis.^45^

### Altered gene expression and regulatory landscape with the 17q21.31-inv

**Figure 5.**
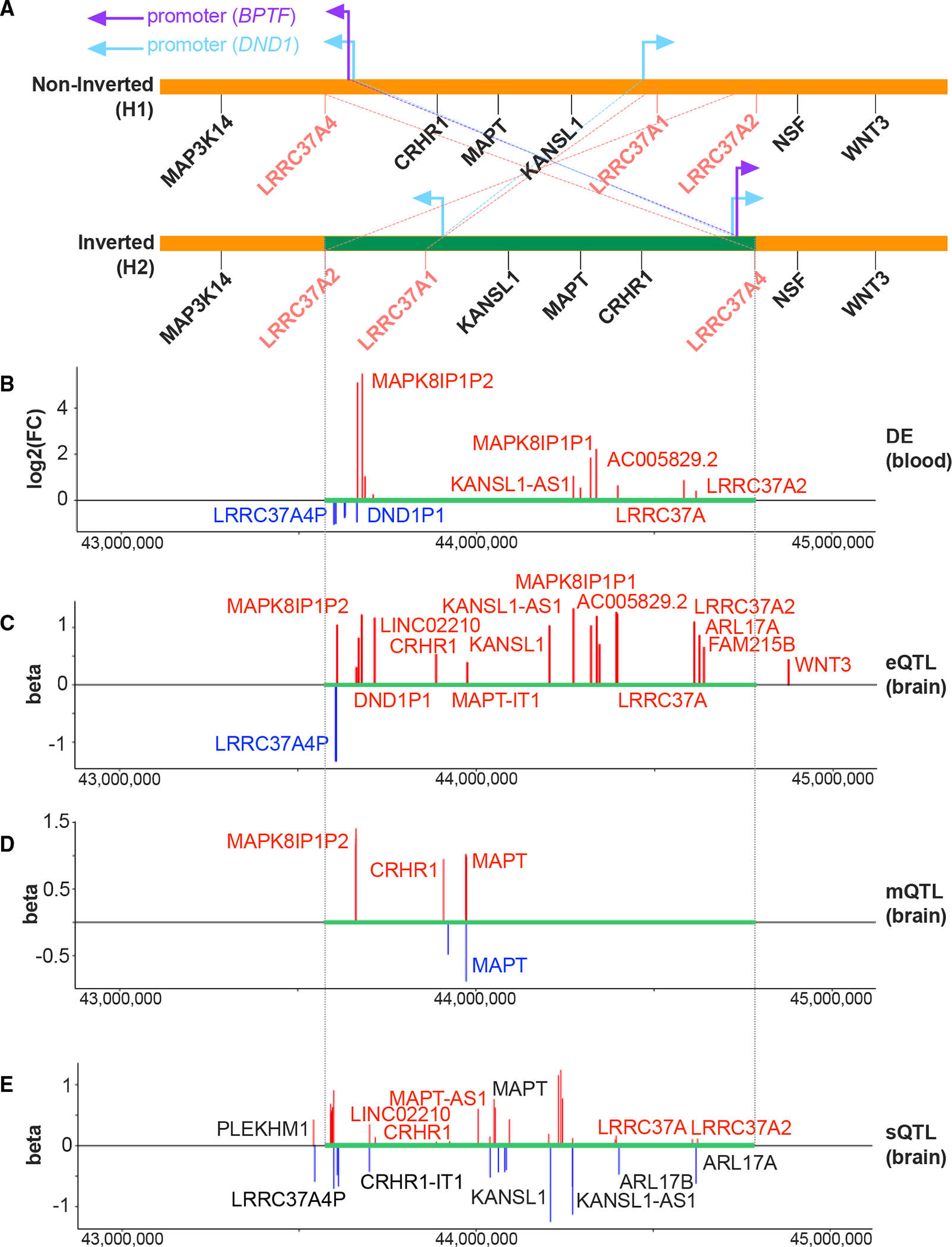
Gene regulatory landscape of the 17q21.31-inv region Plots are aligned based on the base pair position. (A) Schematic plot of the 17q21.31-inv region (in green), adapted from Bekpen et al.^49^ The arrows indicate transcription directions of acquired novel promoters from *BPTF* and *DND1*. Overlying blue bands illustrate regions of segmental duplications (copy-number polymorphisms [CNPs]), with numbers denoting the lengths. (B) Differential expression (DE) of genes comparing inversion carriers with non-carriers. The genes denoted in blue or red represent lower or higher expression for the inversion carriers, respectively. (C) The eQTL analysis reveals that the 17q21.31-inv locus is a genetic variant that affects the expression of its target genes that show differences in gene expression between the inverted and non-inverted alleles. (D) The methylation QTL (mQTL) analysis shows that the 17q21.31-inv locus is a genetic variant that affects the methylation of its target genes. There are multiple CpG sites for a given gene. (E) The splicing QTL (sQTL) analysis demonstrates that the 17q21.31-inv is associated with isoform-level transcriptional abundance of its target genes. The genes indicated in black exhibit both higher and lower abundance of different isoforms. See also Table S5.

Using differential expression (DE) analysis, we identified significant differences in gene expression levels between individuals who carry the 17q21.31-inv and those who do not. Similar to prior work,^46^ we also observed downregulated expression of genes for the *MAPT* H2 haplotype at the upstream breakpoint (e.g., a pseudogene: *LRRC37A4P*) together with upregulated expression of genes within and downstream of the inversion (Figure 5, Table S5). Our expression quantitative trait locus (eQTL) analysis further confirmed these findings (Figure 5, Table S5). In addition, we demonstrated that the inversion acts as a molecular QTL for numerous genes in the inversion region, such as *MAPT, KANSL1, CRHR1*, and *WNT3*, along with their corresponding antisense or long non-coding RNAs (lncRNAs) (e.g., *MAPT-IT1, KANSL1-AS1, CRHR1-IT1*). These non-coding RNAs are known to have regulatory functions.^46,47^ Importantly, several of these genes have well-established roles in neurodevelopmental and physiological functions, highlighting the potential impact of the inversion on complex biological processes.^46^ Our study identified several copies of *LRRC37A*, a family of core duplicon genes that have undergone expansion in primates and have acquired novel promoters, enabling their expression in multiple tissues, are among the genes regulated by the inversion. The exact function of these genes remains to be elucidated, but presumably, they play a role in cellular migration, chemotaxis, and immunity.^48,49^ Similar to our findings, evidence from several studies indicates that 17q21.31-inv is significantly associated with the expression levels of *LRRC37A4P*, A2, and A1 in adult samples^9,46,48,50^ and fetal brains.^51,52^ In short, these findings suggest that the inversion has a significant impact on gene regulation. 17q.21.31-inv with balanced rearrangement does not drastically change gene dosage in the same way as do rare pathogenic CNVs but rather involves changes in a remarkably diverse range of gene regulatory activity with downstream impact on normative variation in brain structure and function. Note that there are common CNVs at the chromosomal 17 inversion breakpoints, which could partially explain the effects of the inversion.

## DISCUSSION

This study provides evidence of common inversions associated with brain morphometry. We found that the 2p22.3-inv, 16p11.2-inv, and 17q21.31-inv reached genome-wide significance (p < 5 × 10^−8^). Eight additional inversions reached significance after the Bonferroni correction, among which 6p21.33 and 8p23.1 are nearly at genome-wide significance (p = 2.1 × 10^−6^ and 1.1 × 10^−7^, respectively). These eight inversions are 6p21.33, 8p23.1, 7p14.1, 6q15, 1q32.1, 4q22.1, 14q23.1, and Xq13.2. Furthermore, our findings were generalizable to 3,472 children from the ABCD study, indicating their replicability. We observed a distinct distribution pattern of significant inversion-morphology associations where strength of association varies across the brain with orderly spatial gradients along core developmental axes. Specifically, the 17q21.31-inv was associated with global surface area reductions, especially posteriorly, with the greatest reduction in occipital and anteromedial temporal areas, accompanied by a reversed pattern of modest increases in cortical thickness prominently in the orbitofrontal cortex. Many of the above-mentioned cytobands are hotspots of structural variations and also encompass pathogenic CNVs linked to neuropsychiatric conditions.^53–55^ Inversions appear to have relatively smaller effects than pathogenic CNVs, perhaps because of their balanced rearrangement and thus high prevalence in the normal population.

The 17q21.31-inv region is one of the most dynamic and complex regions of chromosomal rearrangement in the human genome, spanning up to 1.08–1.49 Mbp.^56^ It comprises two major haplotype families, the NI H1 and the I H2, which differ in orientation and gene copy numbers (also known as *MAPT* haplotypes).^57^ We observed a conspicuous correlation of 17q21.31-inv status with multiple morphological phenotypes of the brain, indicating the inversion’s crucial role in cerebral development and maturation. Previous GWASs of brain MRI phenotypes identified associated variants at the 17q21.31-inv region without determining the direction of inversion effects given the absence of inversion calling. However, the inversion effect can be determined because the 17q21.31-inv is perfectly tagged by SNPs. Interrogating the tag SNPs in summary data from previous studies revealed an association between the H2 haplotype and decreased global total surface area and subcortical and intracranial volumes,^21,22,46,58,59^ which is consistent with the direction of effects we reported. Here, we present detailed analyses of individual brain regions and multiple inversions, revealing intriguing spatial gradations of inversion-associated brain maps. For instance, the H2 haplotype is linked to the greatest regional reductions in cortical area within the visual occipital lobe in conjunction with the greatest thickness increases in the orbitofrontal cortex, a region important for reward processing and decision-making.^60^ A number of genes in this inversion are related to neurodevelopment such as *MAPT*, a gene highly expressed during the prenatal stage (Figure 4). The 17q21.31 I allele frequency varies significantly among populations.^7^ This inversion was shown to be the most significant polymorphism for shaping brain morphology in European populations, but it is rare in Asian populations. Therefore, the results cannot simply be extrapolated across populations; thus, there is a need for genetic studies on other populations.

Compared with 17q21.31-inv, which has dramatic effects on both cortical and subcortical morphometry, we observed opposing effects of 2p22.3-inv on cortical thickness and subcortical volume that were negligible on surface area. The expression of local gene *RASGRP3* was found to be highest in the white matter, followed by various subcortical structures comparing all brain regions,^61^ and highest in oligodendrocytes among all cell types.^62^ This is in contrast to the main local genes of 17q21.31-inv, such as *MAPT*, the expression of which was demonstrated to be highest in the cerebral cortex and within neurons.^61,62^

In contrast to the 17q21.31-inv, we noted that the 8p23.1-inv was associated with increased cortical area and reduced thickness, especially frontal and superior temporal areas and perisylvian thickness comprising language-related cortical regions (Figure 2). The 8p23.1 region is the largest known inversion site in humans (~4.5 Mbp) and captures numerous associated signals of diseases and traits,^63^ with the allele frequency of the inversion ranging from 60% in Africans to 20% in Asians.^64^ The inversion is bordered by the *microcephalin* (*MCPH1*) gene, which is involved in the development of microcephaly and predominantly affects the frontal lobes.^65^

We also found notable associations between the 16p11.2-inv and brain morphometry, particularly the subcortical volumes. The 16p11.2-inv contains *NPIPB* duplicated genes. The *NPIP* gene family showed signatures of positive selection as one of the most rapidly evolving gene families.^66^ Most chromosomal rearrangements are disease causing and are under strong negative selection, but several human expanded gene families (core duplicons) have been shown to be positively selected, potentially harboring genes with selective advantages.^17,67^ *NPIP* functions are unclear but may be immune related.^17^ Prior research has shown 16p11.2 CNVs to be associated with global brain volumes (e.g., duplication carriers tend to have smaller volumes), including total intracranial and gray and white matter volumes.^68–70^ Regional variations associated with 16p11.2 CNVs have also been reported in cortical (e.g., insula/visual cortex) and subcortical structures (e.g., basal ganglia, caudate, and hippocampus),^68–70^ consistent with some of our findings with inversions in this region. These rare pathogenic CNVs show a larger effect than the effect of our inversion in the same 16p11.2 region. Interestingly, the distal CNVs and the inversion (also located distally at 16p11.2) showed the largest associations with reductions of basal ganglia volume among the brain structures investigated.^71^

The 17q21.31, 8p23.1, 16p11.2, and 6p21.33 regions associated with brain morphology here were also previously reported to be linked to multiple neuropsychiatric disorders. The 17q21.31-inv or its local genes have been associated with neurodegenerative disorders.^24,25,72^ 8p23.1-inv was associated with increased risk of autism and neuroticism.^20,73,74^ The *MSRA* gene located at 8p23.1 has been linked to bipolar disorder, schizophrenia, autism, and alcohol use disorder.^75^ CNVs at 16p11.2 showed strong association with autism.^76,77^ The 6p21.33-inv is located within the major histocompatibility complex, which has been linked to schizophrenia (e.g., C4A) and mood disorders.^75,78^

Our multiomics analysis for 17q21.31-inv showed intriguing patterns of inversion-associated transcriptomic and epigenomic changes. Similar to prior work,^46^ we also observed downregulated expression of genes for the H2 haplotype at the upstream breakpoint (e.g., a pseudogene: *LRRC37A4P*) together with upregulated expression of genes within and downstream of the inversion. Here, we showed that the inversion is the molecular QTL for many different genes within the inversion region, including several significant genes underlying neurodevelopmental and physiological functions (e.g., *MAPT, KANSL1, CRHR1, WNT3*) and their corresponding antisense or lncRNAs (e.g., *MAPT-IT1, KANSL1-AS1, CRHR1-IT1*). These antisense or lncRNAs could regulate gene expression of their protein-coding genes and isoforms.^46,47^ Further, we observed and replicated the significance and effect direction of several *LRRC37A* copies: core duplicon genes acquired novel promoters and uniquely expanded in primates with unclear gene function, presumably cellular migration, chemotaxis, and immunity.^48,49^ *LRRC37A4P, A2,* and *A1* have been repeatedly shown to be associated with 17q21.31-inv^9,46,48,50^ and have been demonstrated in fetal brains,^51,52^ though with the cautioning that probes can bind to more than one target genes, especially using earlier array technology.^50^ In short, the 17q.21.31-inv with balanced rearrangement does not drastically change gene dosage in the same way as do pathogenic CNVs but rather involves changes in a remarkably diverse range of gene regulatory activity, with downstream impact on normative variation in brain structure and function.

In conclusion, this study demonstrates that inversion polymorphisms are in the genomic hotspots of neuropsychiatric loci and are associated with brain morphology. In addition to other genetic variants, we underscore that inversion polymorphisms are important contributors to phenotypic diversity pertaining to brain development and disorders.

### Limitations of the study

A key limitation of the present study was ethnicity, owing to the inversion-calling tool *scoreInvHap* that relies on an LD reference panel built on the European population.^6^ Also, *scoreInvHap* predictions are based on limited genotypes, leading to variable reliability of calling results for the inversions with more genotypes. More neuroimaging data are needed from non-European participants for the development of LD reference panels and inversion-calling algorithms that can be applied to other populations. In addition, the array-based approach that this study employed was only capable of detecting larger inversions of specific LD patterns. These issues may be addressed in the future by using other tools, especially novel sequencing-based methods.^79^

## STAR★METHODS

### RESOURCE AVAILABILITY

#### Lead contact

Further information and requests should be directed to and will be fulfilled by the lead contact, Chi-Hua Chen (chc101@ucsd.edu).

#### Materials availability

This study did not generate new unique reagents and physical samples. All computational results of this study are described below in the Data and code availability section.

#### Data and code availability

- The individual-level raw data used in this study can be obtained from UK Biobank (https://www.ukbiobank.ac.uk/) under accession number UKB: 27412 and Adolescent Brain Cognitive Development (ABCD) study (https://abcdstudy.org). Data used in the preparation of this article were obtained from the Parkinson’s Progression Markers Initiative (PPMI) database (https://www.ppmi-info.org/access-data-specimens/download-data/). Please refer to ppmi-info.org for up-to-date information on the study. We made use of publicly available software and tools.
- This paper does not report original code.
- Any additional information required to reanalyze the data reported in this work paper is available from the lead contact upon request.

### EXPERIMENTAL MODEL AND SUBJECT PARTICIPANT DETAILS

#### UKB:

For discovery, genomic, imaging and demographic data were extracted from the UK Biobank (UKB) population cohort, under accession number 27412.^81–84^ The composition, set-up, and data gathering protocols of the UKB have been extensively described elsewhere.^81–83^ Quality control (QC) of imaging and demographic data were detailed in our previous work.^28^ In brief, individuals with ICD10 diagnosis of a neurological or mental disorder were excluded, as well as those with bad structural scan quality based on their age- and sex-adjusted Euler numbers.^85^

#### ABCD:

We conducted an additional analysis to see whether results generalize to a neurodevelopmental cohort. Genomic and phenotype data of children were collected from the Adolescent Brain Cognitive Development (ABCD) study release 3.0 (abcdstudy.org).^86,87^ The database and QC steps were the same as described in our previous work.^28^ In short, those with difficulties to communicate in English, comply with the protocol, or complete a baseline MRI scan were excluded.

In this study, we only included those of European ancestry, because our inversion calling tool *scoreInvHap* relies on its reference haplotypes built from European individuals in the 1000 Genomes (1KG) Project,^6^ and there is a low prevalence of certain inversion genotypes (e.g., 17q21.31-inv) in non-European populations.^39^ Our final sample included 34,720 participants from UKB (age range: 45.13–81.83 years, male-female ratio of 0.9), and 3,472 children from ABCD (age range: 8.92–11.00 years, male-female ratio of 1.1) (Table S2).

### METHOD DETAILS

#### Genotype data

We used UKB Version 3 release and ABCD release 3.0 of imputed genotype data and removed individuals with more than 10% missingness, as well as SNPs with more than 5% missingness, failing the Hardy-Weinberg equilibrium test at *p* = 1e-6 or with minor allele frequencies (MAF) below 0.01.^28^ In this study, we also added imputed X chromosomal data with the same QC steps.

#### Inversion calling

We included 18 out of the 21 human chromosomal inversions (Table S3) that can be studied by the development version of *scoreInvHap*, a tool to classify inversion status from individual SNP data,^6^ based on a selection criterion that these inversions are classified as experimentally validated inversions in the Human Polymorphic Inversion Database (http://invfestdb.uab.cat/)^34^ or present in the list of human inversions recently categorized from the Human Genome Structural Variation Consortium (HGSVC).^4^

The inversion regions of the target genotype data were imputed following the recommended *imputeInversion* pipeline to achieve desirable numbers of SNPs for dense genotype information within the inversion regions. Imputed genotype data were then analyzed by *scoreInvHap* implemented in R 4.0.2. A small percentage of subjects failed inversion calling and did not have inversion genotypes (<1%), and therefore were removed from subsequent analyses.

The built-in inversion genotype classifier was derived from 503 reference individuals with European ancestry in the 1KG. Classification is done for each inversion region separately. The classifier utilizes the frequency, f(s), of an SNP genotype (0,1 or 2) present in a haplotype-genotype group (e.g., inversion-genotype groups [NN, NI, or II]), which was pre-calculated in the reference genomes. The *scoreInvHap* then classifies the haplotype-genotype group K of a new individual based on similarity scores $\left(H_{k}\right)$, calculated as the weighted sum of the above frequencies across all the SNPs within the inversion region $\left(SNP_{i}\ldots SNP_{L}\right)$, where the weights are constructed by the maximum LD between SNP_i_ and the haplotype groups $\left(\rho_{i}^{2}\right)$ and by the certainty (posterior probability) of the imputed SNP_i_ genotype $\left(p_{i}(t)\right)$. The inferred haplotype-genotype group of each individual is assigned to the one with the maximum similarity score among all the groups, denoting the biallelic inverted (I) or non-inverted (N) status as NN, NI or II (see details in Ruiz-Arenas et al., 2019).^6^

$$H_{k}=\frac{\sum_{i=1}^{L}\sum_{s_{i}=0,1,2}p_{i}\left(t\right)\cdot f_{ki}\left(s_{i}\right)\cdot \rho_{i}^{2}}{\sum_{i=1}^{L}p_{i}^{2}}$$

We expanded the list of inversions based on tag SNPs (LD r^2^ > 0.9) in the European population that were reported by Giner-Delgado and colleagues.^19^ Genotypes of 18 additional inversions were called based on inverted (O2) alleles.

#### X-linked dosage compensation

For the Xq13.2-inv, we took account of X-linked dosage compensation (DC) due to random inactivation of one of the X chromosomes in female cells, to balance allele dosage differences in X-linked genes between sexes.^88^ Owing to limited sample size and study power, we could not explicitly determine the presence of DC for all of the phenotypes. Therefore, we conducted association analyses on Xq13.2-inv with both full DC and no DC, coding the inversion genotypes in male subjects as {0,2} and {0,1}, respectively. The results were largely similar under the two extreme conditions from our sensitivity analysis.

#### MRI data and atlases

For UKB, T1-weighted MRI scans were collected from three scanning sites throughout the United Kingdom, all on identical Siemens Skyra 3T scanners.^83^ The standard “recon-all -all” processing pipeline of Freesurfer v5.3 was applied to perform automated surface-based morphometry segmentation.^38^ For ABCD, MRI scans were performed and harmonized across 21 sites and three scanner manufacturers (Siemens Prisma, General Electric 650, and Phillips).^89^ Details on image processing were detailed in previous publications.^90^

For cortical phenotypes, we adopted two genetically-informed atlases, including 12 regions for surface area and 12 for cortical thickness, and 2 global measures of total surface area and mean thickness (Table S1). These atlases have been previously developed by our group, using a data-driven fuzzy clustering technique to identify parcels of the human cortex that are maximally genetically correlated based on the MRI scans of over 400 twins.^32,33^ The subcortical structures were parcellated based on the widely used *aseg* atlas,^38^ including volumes of 20 regions and one global measure of estimated intracranial volume (Table S1). We combined measures of each phenotype across both hemispheres, in view of largely bilateral symmetry of genetic patterning demonstrated in our previous work.^28,91^

### QUANTIFICATION AND STATISTICAL ANALYSIS

#### Genomic structural equation modeling

Previously we explored the joint genetic architecture underlying cortical phenotypes.^28^ We performed genomic structural equation modeling (SEM) on the GWAS summary statistic data of the 12 area and 12 thickness phenotypes separately, which estimates the genetic covariance structure of complex traits. The two-stage analysis includes: 1) estimating the empirical genetic covariance matrix and its associated sampling covariance matrix, and 2) structural equation modeling with user-defined parameters to minimize the discrepancy between the model-informed and empirical genetic covariance matrices.^92^ We did not need to pre-residualize for global effects as they were reflected in the common latent factors.

We used exploratory factor analysis to determine the specifications in the models. Two-factor models were found to be optimal with both the highest cumulative variance explained (0.665 for areas and 0.733 for thicknesses) and lowest number of latent factors. The confirmatory factor analysis demonstrated good model fit for both the area (χ^2^(48) = 1033.55, Akaike Information Criterion (AIC) = 1093.55, comparative fit index (CFI) = 0.99, standardized root-mean-square residual (SRMR) = 0.028) and thickness (χ^2^(48) = 1477.29, AIC = 1541.29, CFI = 0.98, SRMR = 0.042) phenotypes, and outperformed the common factor models for area (χ^2^(54) = 1912.19, AIC = 1960.19, CFI = 0.98, SRMR = 0.041) and thickness (χ^2^(54) = 4603.33, AIC = 4651.33, CFI = 0.95, SRMR = 0.074). The analysis was computed by the R package *genomicSEM*.^92^

#### Quality control for inversion calling

The QC steps of the *ImputeInversion* pipeline after inversion calling involve two steps. We used the default QC parameters to evaluate the reliability of inversion inference for each individual, including the SNP call rates and differences of similarity scores. First, the SNP call rate reports the number of SNPs used in the computation. It reflects the SNP coverage within the inversion regions. *scoreInvHap* recommends at least 15 SNPs for each calling.^6^ Second, a similarity score quantifies how closely the target SNP set resembles the reference genotype of a specific inversion. Classification is good when the highest score is close to 1 and the remaining scores are small, which indicates that the SNP set in the individual is almost identical to one of the reference genotypes and different from the rest, and can be reflected by the differences between the top and second highest similarity scores. The default threshold of difference score is 0.1.^6^

Desirable numbers of SNPs for inversion calling were achieved for all subjects after imputation by the *imputeInversion* pipeline for the first QC criterion. The principal reason for poor inversion calling was the second criterion of similarity scores, indicating that the genotype of samples was more different from the reference haplotype than expected, and/or there were more than one inversion genotypes that the samples resembled. Several inversions have more than two haplotypes^6^ and tend to have lower differences in similarity scores, such as 3q26.1-inv and 7p11.2-inv. Based on the second QC criterion, we excluded 3q26.1-inv, of which less than 75% of calling results passed both QC steps (Table S3), resulting in 17 experimentally validated inversions with good calling quality for the subsequent association analyses.

#### Inversion calling accuracy test

To validate if inversion genotyping from our inversion calling yields the same genotyping as that from a multi-platform sequencing approach, we downloaded subjects from the International Genome Sample Resource (IGSR) built by the 1KG Project, particularly the data contributed by the HGSVC.^93^ We performed inversion calling to classify the 17q21.31-inv status of 17 subjects from the 1KG participants with Hi-C data, using the *scoreInvHap* pipeline. We compared the results with those from the HGSVC data (https://www.internationalgenome.org/data-portal/sample),^94^ which used a multi-platform approach to generate a comprehensive and orthogonally validated set of inversions. They integrated Strand-seq, Bionano optical mapping, and Phased Assembly Variant (PAV)-based variant discovery and identified on average 117 inversions per sample and 316 non-redundant inversions across 32 reference samples from diverse human populations (Table S3).^4^ Although the *scoreInvHap* pipeline is primarily for European data, all of the 17 tested subjects were concordant with the HGSVC inversion genotyping.

We also compared the inversion calling results of five inversions (HsInv0004 (1q31.3), HsInv0040 (2q22.1), HsInv0045 (21q21.3), HsInv0058 (6p21.33), HsInv1053 (7q11.22)) that were included in *scoreInvHap* and with perfect tagging SNPs reported in the European population,^19^ and observed high consistencies between the two methods (accuracy range: 99.39–99.98%, Table S3).

#### Identification of inversion-tag SNPs

To identify SNPs correlated with the inversions, we conducted GWAS with the inversion genotypes as the outcome variables in the UKB data using fastGWA linear regression models.^95^ The Manhattan plots demonstrated that the most significant hits were indeed at the inversion regions as expected (Figure S5). The most significant association signals range from p = 1.29e-303 to p = 4.94e-324 across the inversions. There were no GWAS significant loci (i.e., *p* < 5e-8) outside of the inversion regions, suggesting that there are no existing strong inter- or intra-chromosomal interactions between inversions and SNPs that are outside inversion regions. This set of analyses also confirms the 17q21.21-inv marker SNPs identified previously,^7,39^ given that these marker SNPs were also among the top loci associated with the 17q21.31-inv.

#### Conditional and joint analysis

To determine whether the identified morphology-inversion associations were driven by inversions or the SNPs which the inversions were in LD with, we used conditional and joint (COJO) analysis implemented in Genome-wide Complex Trait Analysis (GCTA),^96^ to select LD-independent SNPs associated with each inversion based on the inversion GWAS described above. Using a stepwise model selection procedure, four SNPs for 2p22.3-inv, ten for 8p21.3-inv, five for 16p11.2-inv and one for 17q21.31-inv were selected. The numbers of major alleles of the COJO-selected SNPs were included as covariates in linear models for each inversion-morphology association analysis where each brain phenotype is the dependent variable and each inversion is the independent variable. We observed that nearly all associations were no longer statistically significant (p < 5.68e-4 for cortical or p < 8.33e-4 for subcortical results), suggesting that the association signals between the COJO-selected SNPs and the inversions were overlapping and the COJO-selected SNPs tagged the inversions.

To evaluate the difference between the genetic effects of the COJO-selected SNPs and inversions, we computed R squared derived from linear regression models where the outcome was the brain phenotype of interest, and the independent variables were the inversions of interest or their COJO-selected SNPs. We observed mostly consistent variance explained by inversions and COJO-selected SNPs in the models, indicating their similar genetic effects.

#### Inversion association analysis

For UKB discovery, prior to inversion association analysis, we regressed out age, sex, scanner site, a proxy of scan quality (FreeSurfer’s Euler number),^85^ and the first ten genetic principal components from each morphometric measure. We also controlled whether or not the individual had a brain diagnosis based on ICD10 diagnostic information collected by the UK Biobank. Similarly for ABCD replication, we regressed out age, sex and scanner site, and removed the imaging data that didn’t pass FreeSurfer QC because Euler numbers were not available. Subsequently, we applied a rank-based inverse normal transformation to the residuals of each measure, ensuring normally distributed input. For the regional measures, the corresponding global measures were also regressed out (i.e., total surface area, mean cortical thickness and intracranial volume for area, thickness and subcortical phenotypes, respectively). This was done to ensure that observed effects were specific to the region of interest, rather than the association with the global measures.

Linear multiple regression models were used to study the associations of inversion genotypes with brain morphometric measures and fluid intelligence scores. The effect of an inversion was considered as additive depending on the number of inverted alleles. We included all 17 inversions as independent variables for analysis of each phenotype.

We removed related individuals prior to the association testing. Using GCTA,^96^ we calculated the pairwise genetic relationship matrix (GRM) based on genome-wide autosomal variants, and removed one related individual from pairs (N = 859 for UKB, and N = 1,850 for ABCD) with an estimated GRM greater than 0.1, which indicates relatedness closer than third cousins. We also removed subjects with failed inversion calls, resulting in 33,018 UKB and 3,472 ABCD participants.

To consider potential correlation between phenotypes, we applied matrix spectral decomposition (matSpD) to determine the effective number of independent phenotypes (*t*_e_),^36^ using correlation matrices of cortical and subcortical measures. Statistical significance was then defined by Bonferroni correction for multiple comparisons (p < 0.05/*t*_e_).

#### Generalization analyses

We assessed the correspondence between the UKB and ABCD results by computing correlation coefficients between the beta estimates of all nominally significant (p < 0.05) inversion identified in the inversion association analysis in UKB. We also used binomial tests to evaluate the consistency of effect directions between UKB and ABCD results by calculating beta sign concordance rate. P-values <0.05 indicate significant effect size correlations and consistency in the direction of associations between the discovery and replication results.^28^ Further details of these approaches to assess replicability can be found elsewhere.^28,97,98^ Note that although using the approaches to test replicability, we use the term ‘generalization’ rather than ‘replication’ between UKB and ABCD to present the results, given that the two cohorts are not in the same age range.^28^

#### BrainSpan Developmental Transcriptome

To explore developmental changes in expression of inversion-related genes, we listed genes at the studied inversion regions based on positions in the UCSC Genome Browser (https://genome.ucsc.edu/). The expression levels in RPKM (reads per kilobase of transcript per million reads mapped) of listed genes were extracted from the BrainSpan Developmental Transcriptome,^29^ and presented as the mean for each inversion at every developmental age through a heatmap.

#### Gene expression analysis of the 17q21.31-inv

To investigate whether inversion genotypes are associated with gene expression changes, genotype and transcriptomic data from blood samples of 187 healthy (age range: 30.60–83.70, mean 60.88 (SD: 11.35) years) participants were downloaded from the Parkinson’s Progressive Marker Initiative (PPMI).^80^ Their 17q21.31-inv status was called following the *scoreInvHap* pipeline as described above. Homozygous inverted subjects (II) were combined with heterozygous subjects (NI) to form the inverted cohort. Differential expression (DE) analysis was performed by the *Seurat* package for R,^99^ comparing the inverted and non-inverted cohorts (II and NI versus NN). Batch effects were removed by *BEER* script for R.^100^ Significant DE genes were defined as FDR <0.05.

#### Molecular QTL analysis of 17q21.31-inv in brain tissues

To test whether the 17q21.31-inv is the eQTL or methylation quantitative trait loci (mQTL) accounting for gene expression changes, we used a tag SNP, rs1396862, to infer 17q21.31-inv status, and to query its eQTL and mQTL results provided in the data resources from summary-based mendelian randomization (SMR).^101^ The default QTL p value threshold of 5e-8 was used. The brain eQTL and sQTL summary data has a sample size of 2865 brain cortex samples from 2443 individuals of European ancestry.^102^ The brain mQTL summary data^98^ has an estimated effective sample size of 1160 derived from a meta-analysis of ROSMAP,^103^ Hannon et al.^104^ and Jaffe et al.^98,105^

## Supplementary Material

1

2

3

4

## Figures and Tables

**KEY RESOURCES TABLE:** 

REAGENT or RESOURCE	SOURCE	IDENTIFIER

Deposited data		

UKB demographic, clinical, genotype and neuroimaging data	UK Biobank version 3	https://www.ukbiobank.ac.uk/
ABCD demographic, genotype and neuroimaging data	ABCD data repository	https://nda.nih.gov/abcd/
BrainSpan developmental brain transcriptomic data	BrainSpan (Tebbenkamp et al.)^29^	http://brainspan.org/static/download.html
Human inversion data	Human Polymorphic Inversion Database (Martínez-Fundichely et al.)^34^	http://invfestdb.uab.cat/
Human inversion data	Human Genome Structural Variation Consortium (Ebert et al.)^4^	https://www.internationalgenome.org/human-genome-structural-variation-consortium
PPMI DNA and RNA sequencing data	Parkinson’s Progression Markers Initiative (PPMI) database (Simuni et al.)^80^	https://www.ppmi-info.org/access-data-specimens/download-data/

Software and algorithms		

R 4.0.2	R	https://cran.r-project.org/
*scoreInvHap* development version	Bioconductor	https://bioconductor.org/packages/release/bioc/html/scoreInvHap.html/
imputeinInversion scripts	Github	https://github.com/isglobal-brge/imputeInversion
*seurat* version 4.1.0	GIthub	https://github.com/satijalab/seurat
GCTA version 1.93.2 beta Linux	Yang Lab	https://yanglab.westlake.edu.cn/software/gcta/
SMR version 1.03	Yang Lab	https://yanglab.westlake.edu.cn/software/smr
Genome table browser	UCSC Genome Browser	https://genome.ucsc.edu/cgi-bin/hgTables

## Highlights

- Chromosomal inversion polymorphisms are associated with human brain morphology
- Inversions are in the genomic hotspots of neuropsychiatric loci
- The 17q21.31 inversion is linked to altered regulation and expression of its local genes
