# Supplementary material for: Chromosomal inversion polymorphisms shape human brain morphology
Source: Cell Rep. Author manuscript; Available in PMC 2023 Sep 19. (PMC10508191; doi:10.1016/j.celrep.2023.112896)
Supplement: 1 [file NIHMS1928249-supplement-1.pdf]

Cell Reports, Volume *42*

Supplemental information

Chromosomal inversion polymorphisms shape human brain morphology

Hao Wang, Carolina Makowski, Yanxiao Zhang, Anna Qi, Tobias Kaufmann, Olav B. Smeland, Mark Fiecas, Jian Yang, Peter M. Visscher, and Chi-Hua Chen

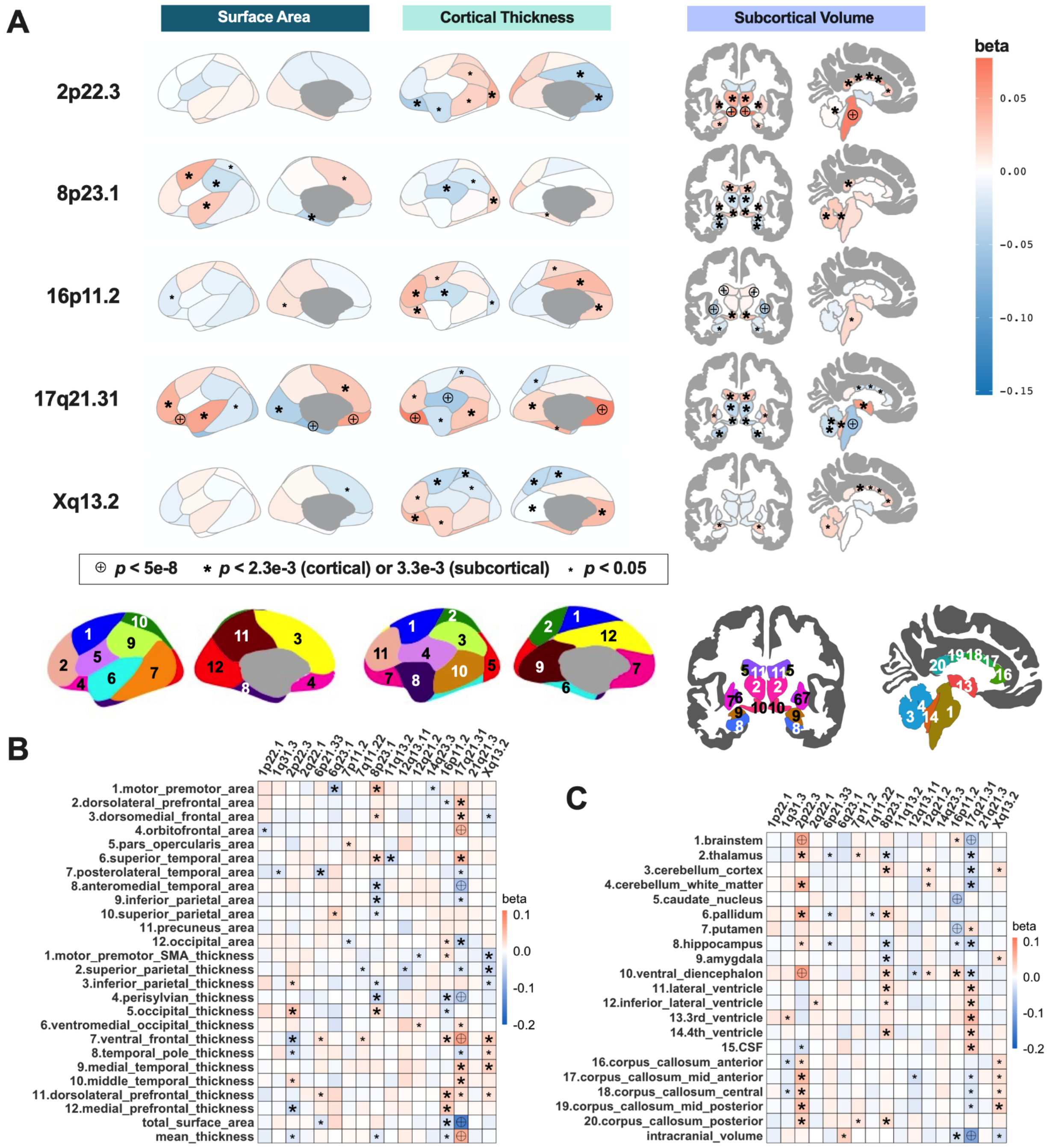

Figure S1. Genetic effects of inversions on cortical and subcortical structures after adjusting for global brain size. Related to Figure 2.

The global measure (total surface area, mean cortical thicknesses or intracranial volume for area, thickness or subcortical regions respectively) was added in a linear model, together with other covariates during the pre-residualization step to regress out their effects, prior to association analysis. (A) Brain maps highlighting the spatial patterns of genetic associations with

morphometric measures for five significant inversions. The bottom row depicts the brain atlases. Numbering of regions follows labels shown on the heatmaps lower panels. (B-C) Heatmaps of associations colored according to the beta coefficients of the regression models for all comparisons (left: cortical; right: subcortical structures). Labels denote nominal (small asterisks), Bonferroni-corrected (big asterisks) and genome-wide significance (encircled crosses). Multiple linear regression models were used. Boxes are colored according to regression coefficients (beta), and labeled where association was nominally (small asterisks, $p < 0.05$), statistically (big asterisks, $p < 3.3e\text{-}3$) or genome-wide (encircled crosses, $p < 5e\text{-}8$).

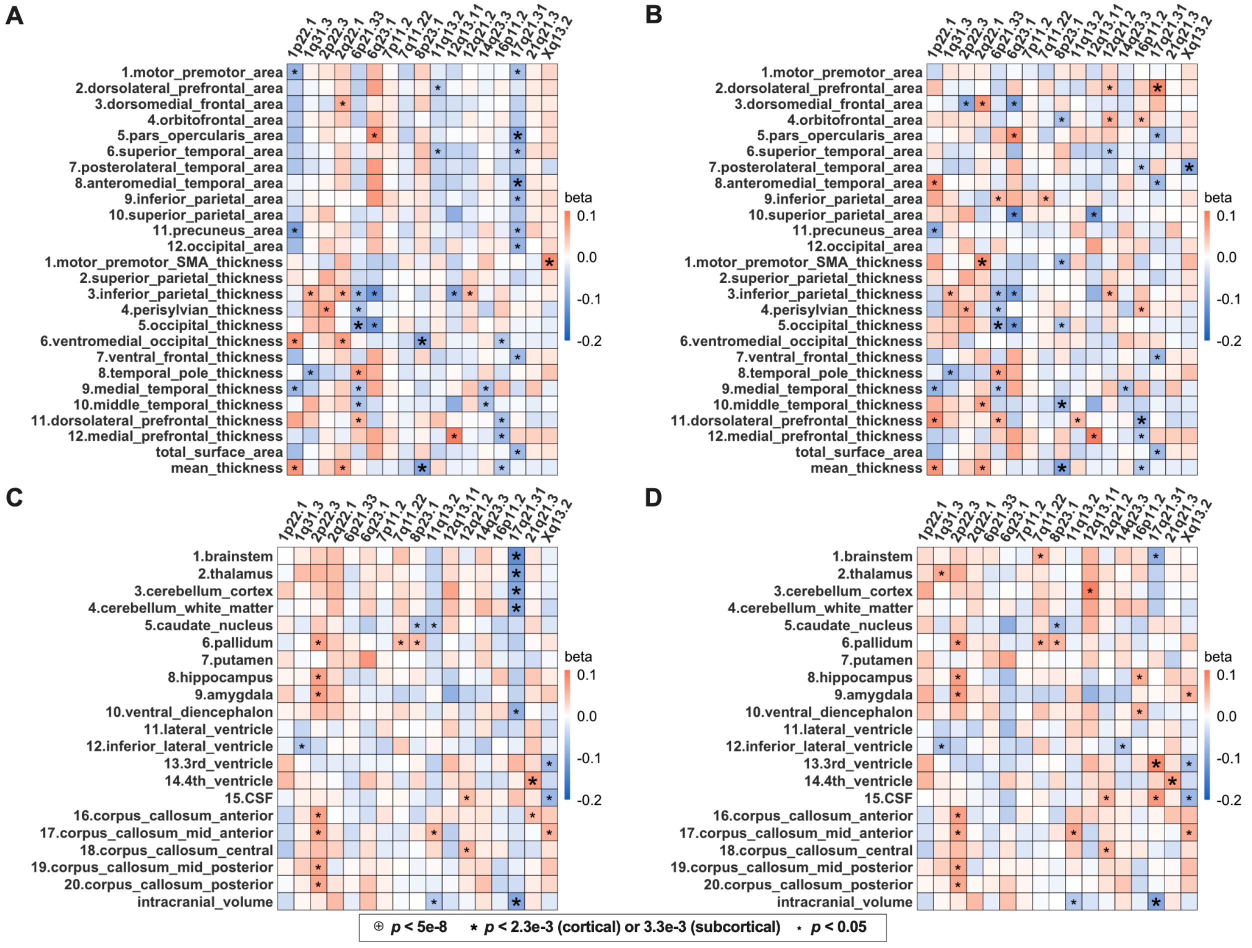

Figure S2. Associations of cortical (A-B) and subcortical (C-D) morphometric measures and inversion genotypes in the ABCD cohort. Related to Figure 2.

Association results after adjusting for global brain size are shown in B and D. Labels denote nominal (small asterisks), Bonferroni-corrected (big asterisks) and genome-wide significance (encircled crosses). Multiple linear regression models were used. Boxes are colored according to regression coefficients (beta), and labeled where association was nominally (small asterisks, $p < 0.05$), statistically (big asterisks, $p < 3.3e-3$) or genome-wide (encircled crosses, $p < 5e-8$).

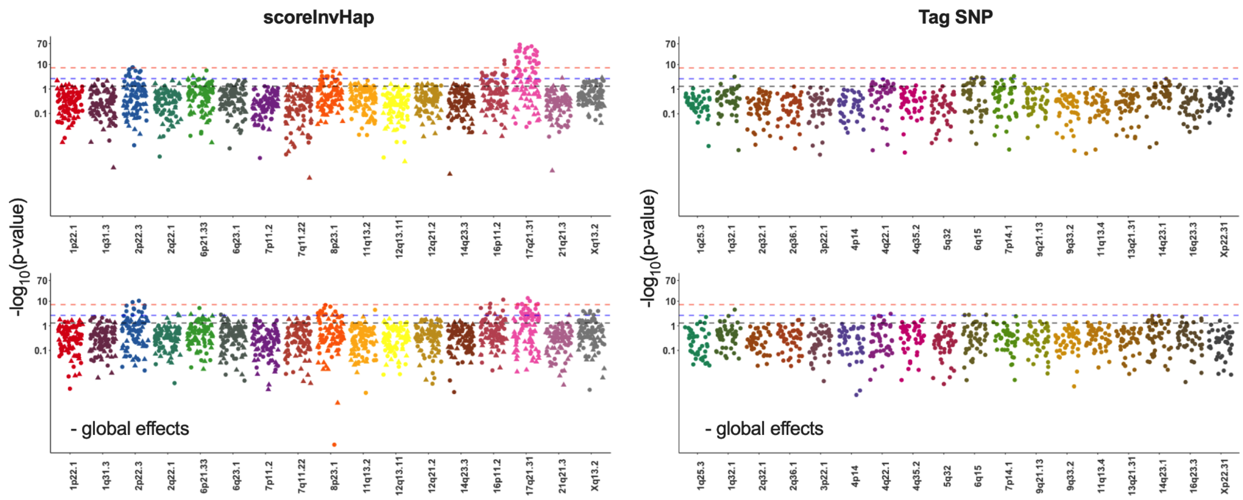

Figure S3. Manhattan plots visualize the association results for each individual inversion-morphology pair with and without adjusting for global measures, using both scoreInvHap (left panel) and tag SNP (right panel) approaches. Related to Figure 3.

The dotted lines indicate the significant levels, ranging from genome-wide, Bonferroni corrected to nominal significance. Results after adjusting for global brain size are shown in the bottom plots.

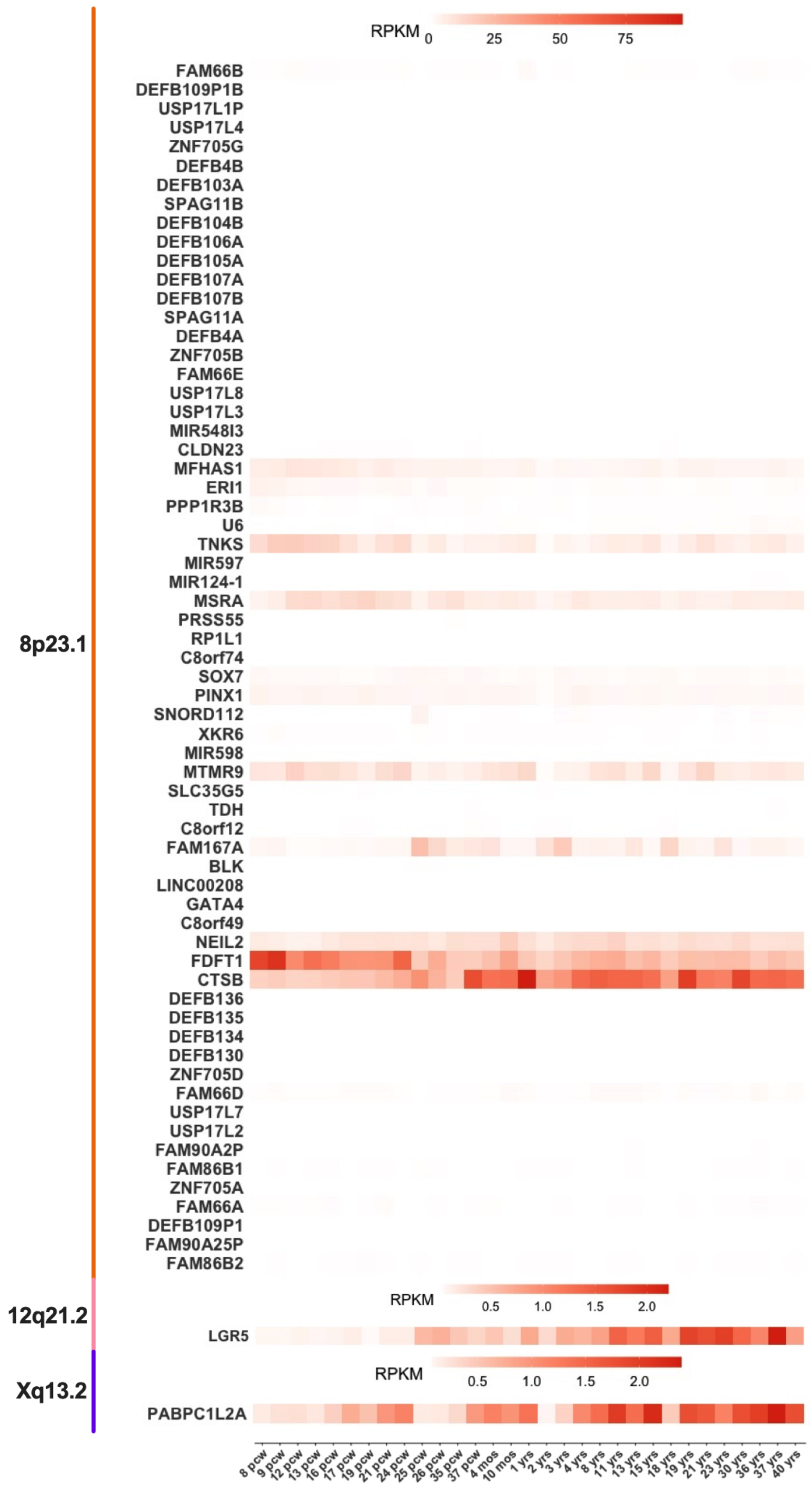

Figure S4. Developmental expression of individual genes within the 8p23.1-inv, 12q21.2-inv and Xq13.2-inv. Related to Figure 4.

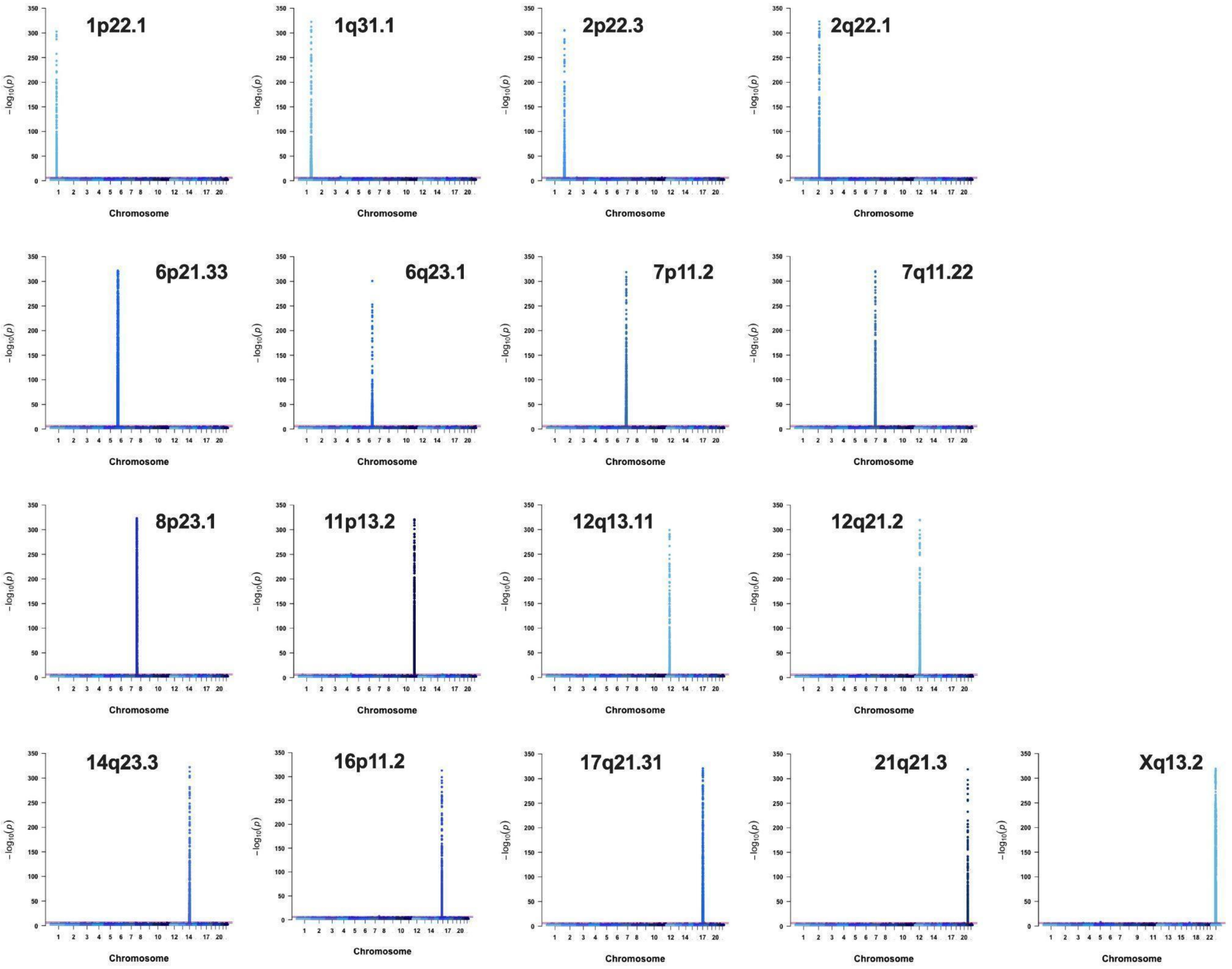

Figure S5. Manhattan plots of GWAS for inversions. Related to STAR Methods.

To identify genetic variants correlated with the inversions, we conducted GWAS with the inversion genotypes as the outcome variables using fastGWA. The Manhattan plots demonstrated that the most significant hits were indeed at the inversion regions as expected. SNPs were liberally clumped in PLINK (LD $r^2 = 0.8$, distance = 250kb).

Table S1. Phenotypes of brain morphometry. Related to STAR Methods.

Cortical Phenotypes (gClust atlas[1,2])		Subcortical Phenotypes (aseg atlas[3])
Surface area	Thickness	Volume
**Frontal lobe**	**Frontal lobe**	SV1. brainstem
SA1. motor-premotor	CT1. motor-premotor-SMA	SV2. thalamus proper
SA2. dorsolateral prefrontal	CT11. dorsolateral prefrontal	**Cerebellum**
SA3. dorsomedial frontal	CT12. medial prefrontal	SV3. cortex
SA4. Orbitofrontal	**Parietal lobe**	SV4. white matter
SA5. pars opercularis	CT2. superior parietal	**Basal ganglia**
**Temporal lobe**	CT3. inferior parietal	SV5. caudate nucleus
SA6. superior temporal	**Perisylvian cortex**	SV6. pallidum
SA7. posterolateral temporal	CT4. perisylvian	SV7. putamen
SA8. anteromedial temporal	**Occipital lobe**	SV8. hippocampus
**Parietal lobe**	CT5. occipital	SV9. amygdala
SA9. inferior parietal	CT6. ventromedial occipital	SV10. ventral diencephalon
SA10. superior parietal	CT7. ventral frontal	**Ventricular system**
SA11. Precuneus	**Temporal lobe**	SV11. lateral ventricle
**Occipital lobe**	CT8. temporal pole	SV12. inferolateral ventricle
SA12. occipital	CT9. medial temporal	SV13. third ventricle
***Total surface area**	CT10. middle temporal	SV14. fourth ventricle
	***Mean cortical thickness**	SV15. cerebrospinal fluid
		**Corpus callosum (CC)**
		SV16. anterior CC
		SV17. mid-anterior CC
		SV18. central CC
		SV19. mid-posterior CC
		SV20. posterior CC
		***Intracranial volume**

* Global measures. Abbreviations: SA - surface area; CT - cortical thickness; SV - subcortical volume.

Table S2. Demographic features of samples. Related to STAR Methods.

	Excluding related individuals		Including related individuals	
Cohort	**UKB (Discovery)**	**ABCD (Generalization)**	**UKB (Discovery)**	**ABCD (Generalization)**
Total N	34,720	4,820	34,720	4,820
N Related Individuals (GRM>0.1)	859	1,348	-	-
N in Analysis	33,861	3,472	-	-
Mean age (SD)	64.32 (7.49)	9.94 (0.62)	64.33 (7.50)	9.94 (0.63)
Age Range	45.13 - 81.83	8.92-11.00	45.13 - 81.83	8.92-11.00
Sex, N(%) Female	17,621 (52.0)	1618 (46.6)	18,134 (52.23)	2,292 (47.6)

SUPPLEMENTARY REFERENCES

1. Chen, C.-H., Gutierrez, E.D., Thompson, W., Panizzon, M.S., Jernigan, T.L., Eyler, L.T., Fennema-Notestine, C., Jak, A.J., Neale, M.C., Franz, C.E., et al. (2012). Hierarchical genetic organization of human cortical surface area. Science *335*, 1634–1636.

2. Chen, C.-H., Fiecas, M., Gutiérrez, E.D., Panizzon, M.S., Eyler, L.T., Vuoksimaa, E., Thompson, W.K., Fennema-Notestine, C., Hagler, D.J., Jr, Jernigan, T.L., et al. (2013). Genetic topography of brain morphology. Proc. Natl. Acad. Sci. U. S. A. *110*, 17089–17094.

3. Fischl, B., Salat, D.H., Busa, E., Albert, M., Dieterich, M., Haselgrove, C., van der Kouwe, A., Killiany, R., Kennedy, D., Klaveness, S., et al. (2002). Whole brain segmentation: automated labeling of neuroanatomical structures in the human brain. Neuron *33*, 341–355.

4. Giner-Delgado, C., Villatoro, S., Lerga-Jaso, J., Gayà-Vidal, M., Oliva, M., Castellano, D., Pantano, L., Bitarello, B.D., Izquierdo, D., Noguera, I., et al. (2019). Evolutionary and functional impact of common polymorphic inversions in the human genome. Nat. Commun. *10*, 4222.
